# Letter to Editor

**Published:** 2015-09

**Authors:** Abdolaziz Haghnegahdar, Shoaleh Shahidi

**Affiliations:** 1 Dept. of Oral & Maxillofacial Radiology, School of Dentistry, Shiraz University of Medical Sciences, Shiraz, Iran.; 2 Biomaterial Research Center, Dept. Oral & Maxillofacial Radiology, School of Dentistry, Shiraz University of Medical Sciences, Shiraz, Iran.

## Dear Sir

Beside its scientific aspects, this letter to editor is a memory letter to remind the deceased great mentor, Dr.Eisa Mozaffari, who introduced a really non-invasive technique to perform sialography which is apparently much safer and more comfortable for the patients. Despite great improvement in MRI and CT scan imaging of salivary glands, sialography is still a popular and trustable method in diagnosis of many problems of salivary glands including ductal system integrity.

Sialography procedure can be divided in to technical phase and diagnostic phase. In technical phase, the orifice of the gland duct should be located and dilated for entrance of contrast material into the ductal and glandular system. After introducing the contrast material, imaging and interpretation is possible. In sialography a” Robinof probe” is used to dilate the orifice of duct. This metallic rigid tool may damage and tear the duct and the surrounding tissues. The ductal orifice must be adapted to this non-resilient object. Rough handling, especially in fine hairy ducts may lead to perforations in the ductal wall as happened in many cases. Consideration of sialography as an invasive procedure by some clinicians may be attributed to the probability of this technical hazard. A perforation makes the following steps of sialography impossible or very difficult to perform.

Dr. Eisa Mozaffari introduced his innovation to avoid the probability of ductal perforation. Instead of lacrimal probe, he used gutta percha points as the dilator of ductal orifice. A cone of gutta percha can be leaded into the orifice of duct when it is grasped firmly by a dental plier, such as that is routine in canal obturation. A no#30 cone is selected to start the procedure. The cone is held in the position for a few seconds that causes the orifice to dilate and accommodate to the diameter of the cone. Then a new and larger-diameter cone will replace the previous one. The procedure will continue serially, until the appropriate dilatation is secured. Usually, cone no#50 performs the job. Because of elasticity of gutta percha, cones will adapt to the ductal anatomy and many curvatures may be passed through without any damage. It is important to hold the cone firmly all the time the cone is placed in the duct since it may be evacuated in to duct if grasped loosely.

This innovation has been applied to both parotid and submandibular salivary glands for many years by post-graduate students of Dr. Mozaffari and researchers in Shiraz dental school, Shiraz, Iran with brilliant results. [1-2] This center and probably other centers supervised by his former residents employ this method in their sialographic procedures. The great advantage of this new sialographic approach is its applicability to all types of ducts, including hairy and those having curves near the orifice. Dr.Mozaffari published his intelligent method of sialography in an article in Persian language at Mashhad Journal of Dentistry (1990).

He hoped this method would change the sialography to a safer and more convenient procedure for both patients and operators worldwide. It is of pleasure for authors, trained by Dr. Mozaffari in Dental School of Shiraz University of Medical Sciences, to answer the questions and present images or videos to whom interested in this exciting alternative sialographic approach. 
